# Histology of adipose tissue inflammation in Dercum's disease, obesity and normal weight controls: a case control study

**DOI:** 10.1186/1476-9255-8-24

**Published:** 2011-09-28

**Authors:** Emma Hansson, Henry Svensson, Unne Stenram, Håkan Brorson

**Affiliations:** 1 Department of Clinical Sciences in Malmö, Lund University, Plastic and Reconstructive Surgery, Skåne University Hospital, Malmö, Sweden; 2 Department of Clinical Sciences in Lund, Lund University, Pathology, Skåne University Hospital, Lund, Sweden

**Keywords:** Dercum's disease, adiposis dolorosa, inflammation, chronic pain, adipose tissue, surgical biopsy, histology

## Abstract

**Background:**

Dercum's disease (DD) is characterised by obesity and chronic pain (> 3 months) in the adipose tissue. The pathogenesis of DD is unknown, but inflammatory components have been proposed. In previous reports and studies, an inconsistent picture of the histological appearance of the adipose tissue in DD has been described. The aim of this investigation was to examine the histological appearance of adipose tissue in patients with DD, with particular focus on inflammatory signs.

**Methods:**

Fat biopsies were sampled from painful regions from 53 patients with DD. In 28 of the patients, a control adipose tissue biopsy was taken from a location where the patient did not experience any pain. In addition, fat biopsies were sampled from 41 healthy pain-free obese control patients and 11 healthy pain-free normal weight control patients. The extent of inflammation was evaluated on histological sections stained with haematoxylin-eosin.

**Results:**

There was no statistically significant difference in the extent of inflammation between the biopsies from the painful knee and the biopsies from the non-painful area (p = 0.5), nor between the biopsies from the abdomen, and the biopsies from the non-painful area (p = 0.4), in patients with DD. A statistically significant difference in extent of inflammation was observed between DD and obese control patients regarding the abdomen (p = 0.022), but not the knee (p = 0.33). There were no differences in extent of inflammation between DD patients and normal weight controls (p = 0.81).

**Conclusion:**

The findings suggest that there is an inflammatory response in the adipose tissue in DD. However, this response is not more pronounced than that in healthy obese controls. This contradicts inflammation as the aetiology of DD.

## Background

Dercum's disease (DD) is characterised by pronounced pain in the adipose tissue and a number of associated symptoms. The pain is chronic (for more than 3 months), symmetrical, often disabling [1] and resistant to analgesics [2]. The pathogenesis of DD is unknown, but inflammatory components have been proposed [2-4]. However, laboratory markers for inflammation, such as erythrocyte sedimentation rate (ESR) and C-reactive protein (CRP), are usually normal in the condition [4-17]. However, a few studies have revealed that some of the patients have elevated levels of CRP and ESR. A study from 1937 of 112 women with DD, reported that 66% had an ESR > 15 mm [18]. Moreover, in a study by Herbst and Asare-Bediako [7], 33.4% of the patients with DD had elevated CRP levels and 37.5% elevated ESR levels. However, 38.2% of the patients included in the study had autoimmune disease, such as rheumatoid arthritis and lupus. In the same study 31.2% of the patients had positive titres for antinuclear antibodies (ANA). It is unclear if these patients were among the 38.2% that had an autoimmune disease. Case reports have shown that markers for autoimmune disease, such as rheumatoid factor (RF), antinuclear antibodies (ANA), anticardiolipin antibodies (ACA), perinuclear anti-neutrophil cytoplasmic antibodies (pANCA), cytoplasmic anti-neutrophil cytoplasmic antibodies (cANCA) and antibodies against native DNA, are commonly negative in DD [4,6,11,15]. Regarding blood cytokines, a small study including 10 subjects and 5 controls [19] indicated that macrophage inflammatory protein (MIP)-1β might be lower in patients with DD than in normal controls. Moreover, a trend towards higher levels of interleukin (IL)-13 and levels of fractalkine were detected.

In previous reports and studies, an inconsistent picture of the histological appearance of the adipose tissue in DD has been described. Fat biopsies in different case reports have revealed histologically normal adipose tissue without inflammation [8,10]. However, pathological findings have been described in other studies. Dercum originally considered the most interesting histological finding to be interstitial inflammation of the nerves in the adipose tissue of the painful sites [1,20], which has only been confirmed in one case report [21].

As regards inflammatory signs in the adipose tissue in DD, leukocytes and plasma cells have been detected in two cases [3,22]. In addition, Herbst et al. [19] found multi-nucleated giant (MNG) cells in three of the DD patients (n = 5) and in none of the controls (n = 5). Multi-nucleated giant cells are produced by activated, pro-inflammatory macrophages. However, no differences in number of macrophages could be seen between the patients and the controls.

Other pathological findings in fat biopsies described in DD are increased levels of connective tissue [19,23], fibrolipoma with numerous embryonic vessels, [24], reactive infiltration of fibrotic elements and small angiomas [25], granulomas [16] and capillary microthrombi [26].

The aim of this investigation was to examine the histological appearance of the adipose tissue in patients with DD, with particular regard to inflammatory signs in a larger series of patients, and compare them with healthy, obese, body mass index (BMI)-matched controls and controls with normal BMI.

## Patients and Methods

### Patients

A total of 53 women with adipose tissue pain were recruited to the study. All patients were diagnosed and referred to our clinic by the same consultant in internal medicine. Diagnosis was based on a systematic physical examination on three separate visits. The clinical criteria of the disease used in this study were obesity (BMI > 28) and chronic pain (> 3 months) in the adipose tissue. The disease can be classified as Type I (juxta-articular), Type II (diffuse-generalised) and Type III (nodular) [2]. All of the patients included in this study had Type II DD. As obese healthy controls, 41 healthy women of similar age and BMI as the DD patients were recruited from the patients operated on with abdominoplasty in our clinic. None of the patients had had major weight loss from bariatric surgery or medical weight loss that could have affected the inflammatory variables. As normal weight healthy controls, 11 women with essentially normal BMI (19 to 26) and of similar age as the DD patients were recruited from the patients operated on because of unilateral leg lymphoedema in our clinic. Four had primary lymphoedema and seven secondary lymphoedema following cancer treatment. The mean duration of the lymphoedema was 16 years (median 15 (range 2-50) years). The patients with secondary lymphoedema had been clinically free from cancer for 15 years (median 14 (range 7-24) years) when the biopsies were taken, and hence should not have any effect on the inflammatory variables. The controls had no acute or chronic pain. The patients and controls were given no restriction in medication and no particular advice regarding lifestyle. None of the patients was diagnosed with any other disease that might give rise to an inflammatory reaction. The patients' profile is given in Table 1. There were no differences between the DD patients and the obese controls as regards age (p = 0.37), weight (0.52) or BMI (p = 0.44). There was a difference between the DD patients and the normal weight controls as regards weight (p = 0.01) and BMI (p = 0.004), but not age (p = 0.50). Similarly, there was a difference between the obese controls and the normal weight controls as regards weight (p = 0.001) and BMI (p = 0.042), but not age (p = 0.37). The differences were analysed using the Mann-Whitney test.

**Table 1:** Patient profile (median and range).

Baseline characteristics	Dercum (n = 53)	Obese controls (n = 41)	Normal weight controls (n = 11)
**Age (years)**	51 (22-68)	50 (26-69)	43 (17-73)
**Weight (kg)**	95 (55-140)	91 (55-129)	69 (52-79)
**BMI (kg/m^2^)**	35 (28-55)	34 (28-46)	24 (19-26)

There were no differences between the DD patients and the obese controls as regards age (p = 0.37), weight (0.52) or BMI (p = 0.44). There was a difference between the DD patients and the normal weight controls as regards weight (p = 0.01) and BMI (p = 0.004), but not age (p = 0.50). Similarly, there was a difference between the obese controls and the normal weight controls as regards weight (p = 0.001) and BMI (p = 0.042), but not age (p = 0.37). The differences were analysed using Mann-Whitney test.

### Fat biopsies

Fat biopsies, obtained by surgical biopsy, were sampled from 53 women with DD. Biopsies were taken from painful subcutaneous fat from the abdomen and the knee region. In 28 cases, a control adipose tissue biopsy was taken from a location where the patient did not experience any pain, 21 from non-painful abdomen and 7 from non-painful knee. Fat biopsies were also sampled from the 41 obese control patients' abdomen and knee region and from the 11 normal weight control patients' knee region. All of the biopsies were open surgical biopsies taken in the same way, measuring about 15 × 15 mm.

**Figure 1:**
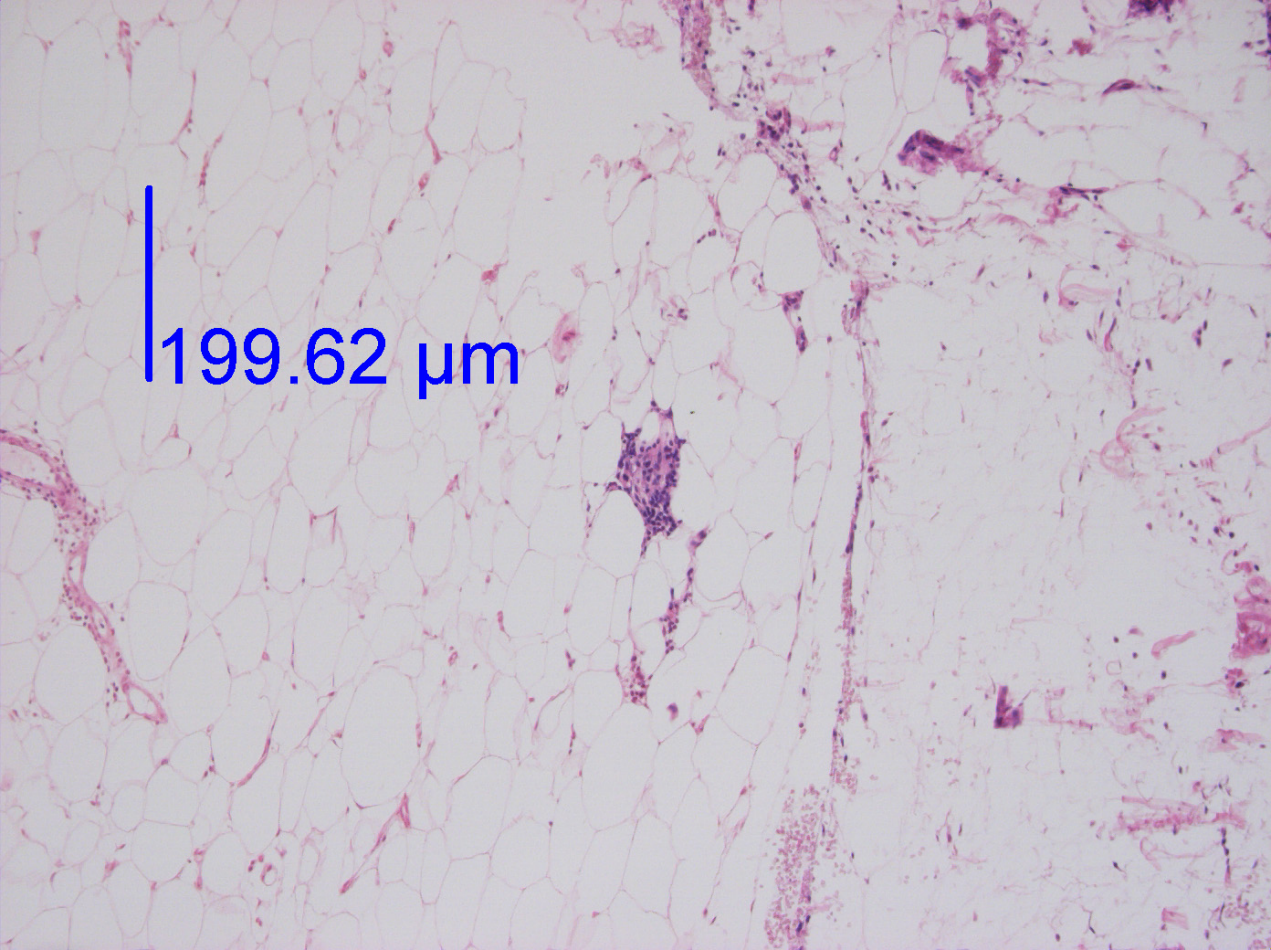
**Low-power image of fat tissue from the knee with an infiltrate of inflammatory cells**. Haematoxylin-eosin staining. The infiltrate is displayed at a higher magnification in Figure 2. The photo was taken with a 10 × objective.

**Figure 2:**
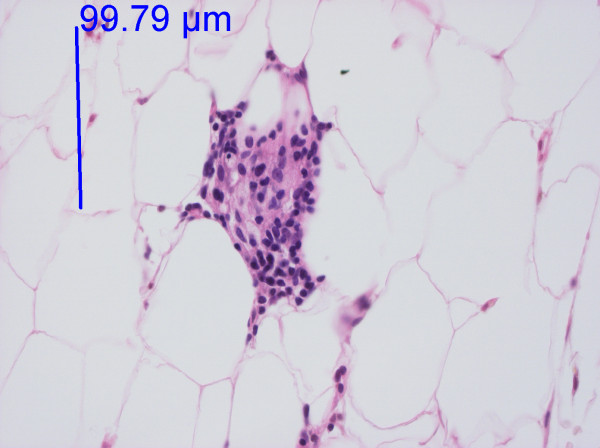
**High-power image of Figure 1**. Adipose tissue from the knee. Haematoxylin-eosin staining. One infiltrate of this size and only a few additional inflammatory cells gave a score of I. Two infiltrates of this size gave a score of II. The photo was taken with a 40 × objective.

**Figure 3:**
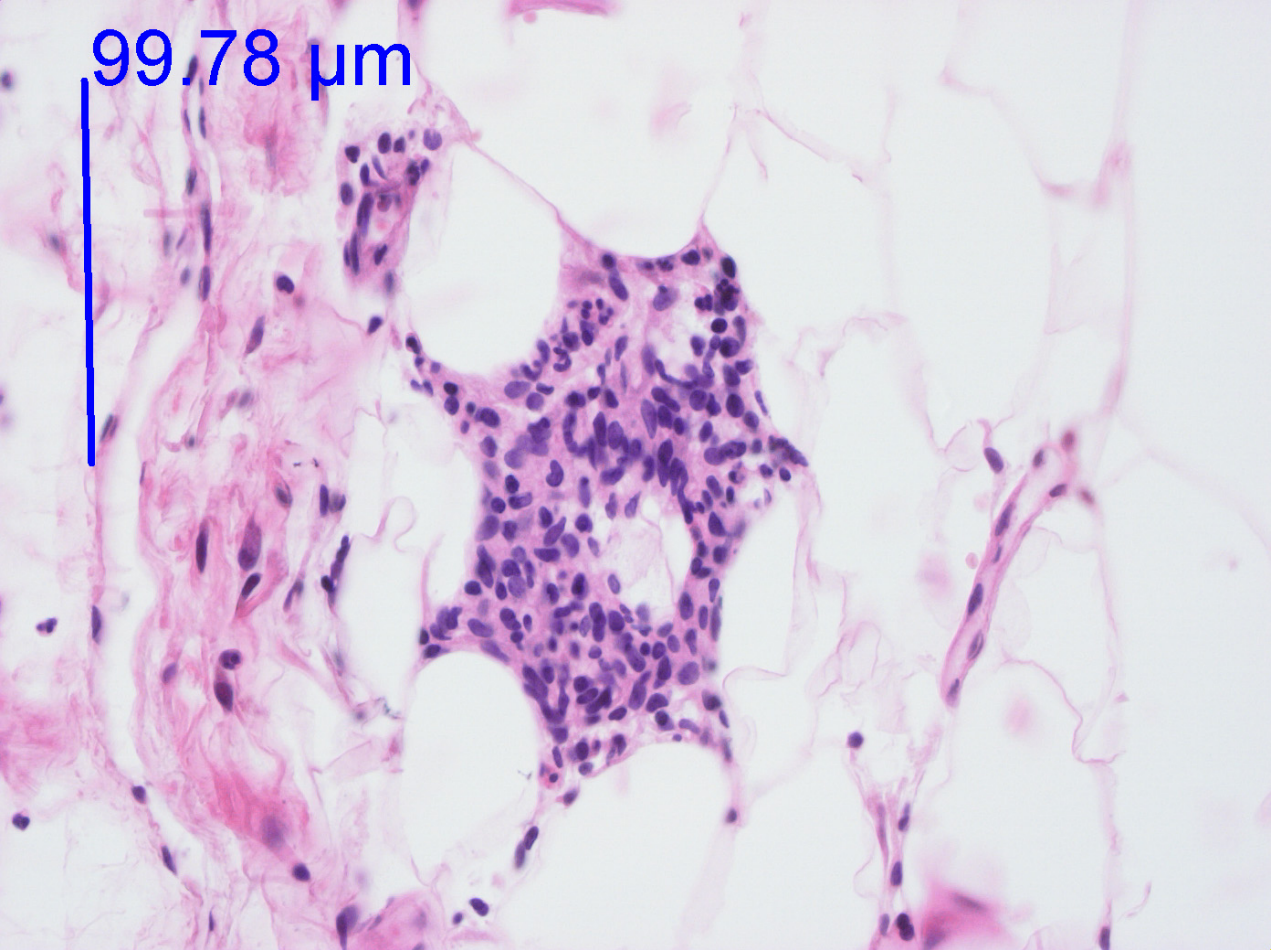
**High-power image of fat tissue from the knee**. Haematoxylin-eosin staining. One infiltrate of this size, larger than that in Figure 2, and a few additional inflammatory cells gave a score of II. Three or more infiltrates of this size gave a score of III. The photo was taken with a 40 × objective.

There were no complications following the biopsies. The biopsies were fixed and transported in a 4% formaldehyde medium and embedded in paraffin. Two consecutive sections were cut from each biopsy and stained with hematoxylin-eosin. The whole sections were examined by the same pathologist (US) in a blinded manner. The inflammatory reaction consisted of lymphocytes, macrophages and possibly some fibroblasts. All the mentioned cells were diagnosed from their appearance in the haematoxylin-eosin staining. The cells were present in aggregates in the fatty tissue as depicted in the figures. A few solitary lymphocytes were also seen. The extent of the inflammatory reaction was evaluated subjectively as described in Figures 1, 2 and 3, taking into consideration number of and size of the inflammatory infiltrates. There is thus a continuum of changes. The inflammatory reactions were given a score between 0 and III, where 0 equalled no inflammation, I slight, II moderate and III pronounced inflammatory reaction. For further explanation, see Figures 1, 2 and 3.

### Laboratory tests

The erythrocyte sedimentation rate (ESR) was measured using the Westergren method, that is 4 parts blood were diluted with 1 part isotonic citric solution. The level of sedimentation was measured after 1 hour. The reference intervals for ESR for Swedish women are < 21 mm up to 50 years of age, and < 30 mm between 51 and 70 years of age [27].

### Ethics

The study was approved by the Ethics of Human Investigation Committee at Lund University (LU 236-89, LU 422-91). All participants gave their written informed consent to participate. The procedures were in accordance with the Helsinki Declaration of 1964.

### Statistics

Values are given as medians and ranges. Histograms were drawn to examine the distribution of the measured factors. The histograms indicated that the measured factors were not normally distributed. Differences in highest inflammatory reaction between biopsies from painful locations and non-painful locations from DD patients were analysed using paired McNemar's test. Biopsies taken from the painful knee region in DD patients were compared to those from the knee region of control patients, using chi-square tests. The same procedure was used for the abdomen. In all cases the highest score for each biopsy was used when comparisons were made. Differences in ESR between the DD patients and the obese controls were analysed using the Mann Whitney test.

## Results

In the DD patients, 75% of the biopsies from painful areas and 71% of the control biopsies from non-painful areas (Table 2) demonstrated an inflammatory reaction (I-III) with lymphocytes and macrophages (Figures 1, 2 and 3). An inflammatory reaction judged as I can be seen in Figures 1 and 2 and II in Figure 3. In the obese controls, 73% of the biopsies demonstrated an inflammatory reaction (I-III) and in the normal weighted controls 45% of the biopsies demonstrated a slight inflammatory reaction (I). Inflammatory reactions in adipose tissue in all groups are summarised in Table 2. Plasma cells were found in very few of the biopsies. There was no difference between adipose tissue from painful and non-painful abdomen (p = 0.4) or knee (p = 0.5).

**Table 2:** Inflammatory reaction (score 0 to III) in fat biopsies and intra- and intergroup differences.

Group	Biopsy	Reaction			
		0	I	II	III
**Dercum**	Painful abdomen (n = 41)	11 (27)	14 (34)	16 (39)	-
	Painful knee (n = 47)	11 (23)	21 (45)	14 (30)	1 (2)
	Non-painful area (n = 28)	8 (29)	14 (50)	6 (21)	-
**Obese controls**	Abdomen (n = 41)	12 (29)	15 (37)	12 (29)	2 (5)
	Knee (n = 41)	10 (24)	13 (32)	16 (39)	2 (5)
**Normal weight controls**	Knee (n = 11)	6 (55)	5 (45)	-	-
**Differences between groups (p-value)**					
Dercum vs. Obese controls - abdomen			0.022		
Dercum vs. Obese controls - knee			0.33		
Dercum vs. Normal weight controls - knee			0.81		
Obese vs. Normal weight controls - knee			< 0.001		
Dercum - painful abdomen vs. Dercum - non-painful abdomen^1^			0.4		
Dercum - painful knee vs. Dercum - non-painful knee^2^			0.5		

^1^n = 21 pairs. ^2^n = 7 pairs. Figures within parenthesis depict percentage of total.

Number of patients (%). Differences in highest inflammatory reaction between biopsies from painful locations and non-painful locations from DD patients were analysed using paired McNemar's test. Biopsies taken from the painful knee region in DD patients were compared to those from the knee region of control patients, using chi-square tests. The same procedure was used for the abdomen. In all cases the highest score for each biopsy was used when comparisons were made.

Furthermore, no differences in extent of inflammation were detected between DD and obese control patients regarding the biopsies from the knee region (p = 0.33, χ^2 ^= 22.2, df = 20). A significant difference in extent of inflammation was observed between DD patients and obese controls patients for the biopsies from the abdomen (p = 0.022, χ^2 ^= 29.3, df = 16).

There were no differences in extent of inflammation between DD patients and normal weight controls (p = 0.81, χ^2 ^= 6.1, df = 10) (knee). However, a difference was detected between obese controls and normal weight controls (p < 0.001, χ^2 ^= 31.4, df = 8) (knee). The differences within and between the groups are summarised in Table 2.

Among the DD patients of 50 years and younger, 26 patients had an ESR of < 21 mm and 2 patients > 21 mm (24 and 38 respectively) (median 11 mm (range, 4-38)). Values were missing from three patients in the younger age group. Among the DD patients over 50 years of age, all but one had an ESR of < 30 mm (median 9.5 (range 4-34)). The patient with a higher value had an ESR of 34 mm. Values were missing from two patients in the older age group. Among the obese control patients of 50 years and younger, all but two had an ESR of < 21 mm, and two patients had values > 21 mm (29 and 90 respectively) (median 11.5 (range 2-90)). Values were missing from 5 patients in the younger age group. Among the obese control patients over 50 years of age, all but two patients had an ESR of > 30 mm (median 15 (range, 1-41)). Values were missing from 8 patients in the older age group. There was no statistical difference in ESR between the DD patients and the obese controls, neither in the age group < 50 years (p = 0.99), nor in the age group > 50 years (p = 0.73).

## Discussion

The strengths of the study are that the same consultant diagnosed DD in all patients and that a control group of healthy obese controls was included. Furthermore, no study has been published with a greater number of patients with DD examined through fat biopsies.

Studies in anatomical pathology as gold standard has been challenged because of the difficulties in reproducibility of histological diagnosis due to inter-observer variation. This can be explained by the fact that interpretive judgement and personal experience have to be used by the pathologist to be able to make a histopathological diagnosis [28]. However, in the present study, the same pathologist judged all the fat biopsies in a blinded fashion, and hence, such factors should be of less influence. A limitation of the present study is that we had no information on the use of over-the-counter analgesics. It is possible that such drugs could have affected the inflammatory reaction in the adipose tissue.

An inconsistent picture of the histological appearance of the adipose tissue in DD has been reported in previous reports and studies. Recently, Herbst et al. [19] found multinucleated giant cells in three patients with this condition. However, when these patients were compared with healthy obese controls, no differences in the inflammatory reaction were seen. In this study, a difference in the inflammatory reaction in the adipose tissue could be seen between patients with DD and healthy obese controls comparing biopsies from the abdomen but not from the knee. In recent years, research has suggested that the adipose tissue in obesity elicits a chronic low-grade inflammatory response that contributes to co-morbidities such as diabetes, increased cardiovascular risk and liver disease [29-31]. The expanded pool of adipocytes is responsible for the increased production and release of inflammatory mediators such as cytokines. An increased density of macrophages has been observed in the adipose tissue of obese subjects [31,32]. This can explain why DD patients and weight-matched healthy obese controls both have an elevated inflammatory reaction and the presence of macrophages in the adipose tissue.

In conclusion, our findings reveal that there is an inflammatory response in the adipose tissue in DD. However, this response might not be more pronounced than that in healthy obese controls. This contradicts inflammation as the aetiology of Dercum's disease.

## Declaration of Competing interests

The authors declare that they have no competing interests.

## Authors' contributions

EH participated in the design of the study, performed the statistical analysis and wrote the manuscript. HS participated in the choice of statistical methods and in the writing of the manuscript. US participated in the design of the study, carried out the histological judgement and contributed to the writing of the manuscript. HB initiated and designed the study, took all biopsies and contributed to the writing of the manuscript. All authors have read and approved the final manuscript.
